# Gender, Heteronormative Attitudes, and Sexual Identity Stability and Change

**DOI:** 10.1007/s10508-025-03301-4

**Published:** 2025-12-02

**Authors:** Alice Campbell, Tony Silva

**Affiliations:** 1 School of Social Sciences, University of Tasmania, Levels 4/5 Social Sciences Building, Churchill Ave., Sandy Bay, Hobart, TAS 7005 Australia; 2 Department of Sociology, University of British Columbia, Vancouver, BC Canada

**Keywords:** Sexual identity, Identity change, Heteronormativity, Homonegativity, Attitudes, Sexual orientation

## Abstract

Changes between heterosexual (straight) and lesbian, gay, bisexual, queer, and questioning (LGBQ) identities have been observed among a substantial minority of people in longitudinal studies. While a robust base of the literature has explored why women are more likely than men to identify as LGBQ, little research has investigated gendered social factors associated with changes in sexual identity—especially changes from an LGBQ identity to straight. To address this gap, we tested associations between preference for heterosexuality, endorsement of conventional family ideology, and changes in sexual identity in women and men. Analyzing data from a large and representative longitudinal study of Australian adults (ages 20–99 years, *M* = 50.63), we found a positive association between the endorsement of conventional family ideology and the odds of changing to a straight identity among LGBQ women but not men. Meanwhile, greater preference for heterosexuality increased the odds of changing to a straight identity among GBQ men but not LGBQ women. The vast majority of those changing to a straight identity had previously identified as bisexual, other, or unsure, with very few people changing from gay or lesbian to straight. Our results indicate that social factors are related to sexual identity change in gendered ways.

## Introduction

Sexual identification is not necessarily static, and evidence from several countries shows that it is common for people to change how they report their sexual identity on surveys. Such changes have been observed across the life course, from young adulthood (Campbell et al., 2020, 2021; Ott et al., 2011; Savin-Williams & Ream, 2007; Savin-Williams et al., 2012) to middle age (Dickson et al., 2013; Mock & Eibach, 2012) and older adulthood (Hu & Denier, 2023). There is some evidence that changes in sexual identity are most prevalent among young women (Mittleman, 2023a). However, any gender differences in the propensity to change sexual identity might depend on directionality. It was recently observed that women are more likely than men to change to a plurisexual identity (an identity which indicates attraction to multiple genders, such as bisexual or pansexual) from a heterosexual or lesbian/gay identity, but less likely than men to change from a plurisexual to a lesbian/gay identity (Lilly et al., 2024).

Although a robust literature exists about sexual identity change (see Srivastava et al., 2022 for a recent review), there remain large gaps in our knowledge about factors which predict sexual identity change. Identification is an embedded process shaped by all levels of social context (Burke & Stets, 2023). Yet, the social factors associated with changes to sexual identity, and how these factors might vary for women are men, are under-explored. This paper helps fill that gap in the literature by using a large sample from the Household, Income and Labour Dynamics in Australia (HILDA) Survey, a large and nationally representative, annual survey of Australia. This paper analyzes whether preference for heterosexuality and conventional family attitudes (among other factors) are significantly associated with sexual identity change toward or away from a lesbian, gay, bisexual, or queer (LGBQ) identity, and whether there are significant interactions between these attitudes and gender.

### Social Factors and Sexual Identity Change

Several factors may shape a person’s propensity to change their sexual identity (e.g., Diamond, 2021; Lilly et al., 2024). While humans are motivated to maintain stable identities, they are also motivated to reduce perceived discrepancies between identity standards (i.e., the meanings attached to an identity) and their other identities and behavior (Burke, 2006). Thus, some people might change their sexual identity to bring it into alignment with their sexual attractions, behavior, or gender identity.^1^ Indeed, experiencing attractions or behaviors socially perceived as incompatible with a particular sexual identity—such as same-sex attractions alongside a heterosexual identity—may lead some individuals to change their identity. Consistent with this framework, changes to sexual identification often occur alongside changes in self-reported attractions (Mittleman, 2023a; Silva, 2019) or partner gender (Hu & Denier, 2023) or following gender transition/affirmation (Davis & Amand, 2014; Dozier, 2005). In addition, there is some evidence that changes away from a heterosexual identity are more common among people who score higher on the personality trait of openness to experience (Lilly et al., 2024), and lower on the cognitive trait of personal need for structure (Preciado & Peplau, 2012).

Beyond individual circumstances and psychology, broad social and cultural forces are arguably important motivators of sexual identity change. According to Stets (2021, p. 304), identity change is a nested phenomenon, “a product of macro, meso, and micro structural and cultural dynamics.” We contend that heteronormativity is among the most important dynamics for understanding changes in sexual identity. Consistent with Ortyl’s (2013) definition, we understand heteronormativity as comprising two dimensions. The first dimension is homonegativity, which involves the privileging of heterosexuality and the marginalization or active fear and dislike of LGBQ people (i.e., heterosexism and homophobia). The second is institutionalized heterosexuality, which involves the privileging of married, monogamous heterosexual couples with children over all other family formations. Heteronormativity thus encapsulates beliefs about what is the natural and moral way to do gender, be sexual, conduct an intimate relationship, and have a family. These beliefs are expressed by individuals, portrayed in the media, and enshrined in legislation and institutions. As such, heteronormativity manifests as both social structure and culture.

Preliminary evidence suggests that the level of heteronormativity in a person’s contexts can facilitate or constrain their adoption of a non-heterosexual identity. For example, individuals experienced more distress when first questioning their sexual identity the greater their exposure to heteronormative messages growing up (Boyer & Lorenz, 2020). Living in a state that recognized same-sex relationships was associated with a higher likelihood of changing from a heterosexual to an LGBQ identity in a large sample of US women (Charlton et al., 2016). Schools, neighborhoods, and social networks are also associated with the likelihood of identifying as exclusively straight (Silva & Evans, 2022). Another study found that young women were less likely to change from a heterosexual to an LGBQ identity if they lived in a rural area with their parents rather than in a metropolitan area with their peers or alone (Campbell, 2023).

Of course, social structures and culture do not only exist “out there” but can be internalized and incorporated into an individual’s own attitudes and values. It is therefore pertinent to examine associations between internalized heteronormativity and sexual identity change. In previous research, high levels of internalized homophobia among Black men who had sex with men were associated with a greater likelihood of changing to a heterosexual identity from a non-heterosexual identity 6–12 months after the initial survey (Phillips et al., 2020). Among a sample of young women identifying as exclusively heterosexual, rejection of institutionalized-heterosexuality norms was associated with an increased likelihood of changing to a mostly heterosexual or bisexual identity (Campbell, 2023). Higher religiosity and political conservatism—both of which are correlated with homonegativity and traditional family ideology (e.g., Perales & Bouma, 2019; Perales et al., 2019; Prusaczyk & Hodson, 2020)—have also been identified as significant predictors of the propensity to change sexual identity. Scheitle and Wolf (2018) found that higher religiosity was associated with a greater likelihood of changing away from a gay or lesbian identity, while Lilly et al. (2024) found that higher religiosity and political conservatism were each associated with lower odds of changing from heterosexual to plurisexual identities.

While ground-breaking in many ways, the above studies either used single-gender samples (Campbell, 2023; Phillips et al., 2020) or failed to test for gender differences in the factors associated with sexual identity change (Lilly et al., 2024; Scheitle & Wolf, 2018). To our knowledge, only two prior studies have examined gender differences in associations between social attitudes/values and changes in sexual identity. Silva (2018, 2019) found that increased religiosity and political conservatism were associated with US women’s—but not men’s—odds of changing from an LGBQ to a straight identity. These findings suggest that there could be gendered constraints on adopting and maintaining an LGBQ identity, shaping the sexual identity trajectories of men and women in distinct ways. This premise is consistent with sociological theories of gender and sexuality, described in the following section.

### Gendered Constraints on Sexual Identity

According to sociological theory, men's homosexuality^2^ is incompatible with most configurations of hegemonic masculinity (i.e., the masculine cultural ideal: Connell & Messerschmidt, 2005; Diefendorf & Bridges, 2020). In many contexts, “real men” are expected to dominate women, including sexually. Gay men violate this expectation and, in doing so, are perceived as threatening the gender order. Homophobic bullying is used to police gender among boys and men, and as such is a key strategy for dealing with this threat (Mittleman, 2023b). As a result, same-sex behavior between men is more stigmatized than same-sex behavior between women, and attitudes toward gay and bisexual men are more hostile than attitudes toward lesbian and bisexual women (Bettinsoli et al., 2020; England, 2016; Yost & Thomas, 2012). Further, men are more homophobic on average than women, especially when the target is a GBQ man (Bettinsoli et al., 2020; Yost & Thomas, 2012). It therefore seems plausible that internalized homonegativity could be a more salient factor in shaping the sexual identities of men than women.

Indeed, a certain amount of sexual contact between women is currently permitted and even encouraged in Western societies such as Australia and the USA—albeit, within tightly policed boundaries. So-called lesbian porn, threesomes between one man and two women, and “straight girls kissing” at college parties are enthusiastically embraced by many heterosexual men (Diamond, 2005; Pham, 2019; Yost & McCarthy, 2012). As Rupp et al. (2014) argued, “bisexual behavior between women has flourished in a variety of societies where women’s same-sex desires and behavior did not pose a threat to the gender order” (p. 216). Crucially, according to these authors same-sex behavior between women is not considered a threat to the patriarchal gender order so long as it perceived as complementing rather than replacing heterosexuality (Rupp et al., 2014). Women’s sexual receptivity to men is central to most configurations of hegemonic masculinity and emphasized femininity (Connell, 1987), and deviations can be harshly punished. When women engage in same-sex behavior that is not performative but rather for their own pleasure, the consequences can include ostracism and disgust from other women, and anger and violence from men (Byron et al., 2017; Hamilton, 2007; Rupp & Taylor, 2010).

Bisexual women in particular can find themselves in a double bind. Stereotyped as unreliable, promiscuous, greedy, and hypersexual (Dyar & Feinstein, 2018; Lahti, 2018; Mize & Manago, 2018), bisexual women are all at once fetishized, objectified, distrusted, and expected to “prove” their bisexuality performatively (Flanders et al., 2017; Whitney, 2001). These dynamics arguably contribute to the higher rates of intimate partner and sexual violence that bisexual women experience compared to lesbian and heterosexual women (Campbell, 2022; Chen et al., 2020). In addition, the negative stereotypes about bisexual women position them (unfairly) as less than ideal mothers (Campbell, 2022). To be clear, there is no evidence that bisexual women are any less loving, warm, or competent as mothers than anyone else. However, the ambient stereotypes surrounding bisexuality mean that some women experience conflict between their sexual identity and their identities as wives and mothers (Budnick, 2016; Tasker & Delvoye, 2015). It therefore seems plausible that stronger endorsement of conventional family ideology could make it psychologically difficult for some women to maintain a bisexual identity, “pushing” them to change to a heterosexual identity.

In a separate but related area of research, psychologists have examined the extent to which biological sex differences may shape sexual behavior, attraction, and identity. Some psychologists argue that women are more likely to experience attraction to multiple genders and to experience shifts in their attractions over time (Diamond, 2009), whereas men are more likely to have attractions to a single gender and are less likely to experience changes to their attractions (Bailey, 2009). Similarly, others have argued that situational, social, and cultural factors affect women’s sexuality more than men’s (Baumeister, 2000). All these differences may, in turn, affect both the likelihood of identifying as LGBQ and the likelihood of changing sexual identities. Social and attitudinal factors, which are the focus of this study, work alongside or in the context of the aforementioned differences psychologists argue shape women’s and men’s sexualities.

### Study Contributions

Despite a robust base of research about how sexual identification and sexual behavior are gendered, little prior work has quantitatively explored whether social factors such as internalized heteronormativity are associated with sexual identity change in gendered ways. This study examines the extent to which preference for heterosexuality and conventional family ideology have gendered outcomes related to sexual identity change. Our review of the extant theory and evidence leads us to expect that endorsement of conventional family ideology will have a stronger constraining effect on the sexual identities of women compared to men. As such, we expect that stronger endorsement of conventional family ideology will be associated with increased odds of changing to a straight identity for LGBQ-identified women, and decreased odds of changing to an LGBQ identity for straight-identified women. In contrast, we expect preference for heterosexuality to have a stronger constraining effect on the sexual identities of men compared to women. As such, we expect that greater preference for heterosexuality will be associated with increased odds of changing to a straight identity for GBQ-identified men, and decreased odds of changing to a GBQ identity for straight-identified men.

## Method

### Participants

We analyzed data from the Household, Income and Labour Dynamics in Australia (HILDA) Survey (Watson, 2021). The HILDA Survey is an ongoing household panel study of a similar design to the US Panel Study of Income Dynamics, the German Socio-Economic Panel, and the British Household Panel Survey. It commenced in 2001 (Wave 1) with a national probability sample of almost 20,000 Australian residents (13,969 of whom were aged 15 + years) from more than 7500 households. The sample was selected via a multi-stage clustered stratified design and topped up in 2011 with an additional 4,009 respondents from 2153 households selected using the same procedures. The HILDA Survey collects data annually via interviews and a self-completed questionnaire (SCQ), which all participants aged 15 years and older are invited to complete. While some topics are covered in the survey every year (e.g., labor market and health outcomes), other topics are included on a rotating 4-yearly basis (e.g., various attitudinal measures). Our key variables were all taken from the SCQ component of the HILDA survey, which has a response rate of approximately 89% in each wave. We confirm that this study was granted an exemption by the appropriate institutional research ethics committee.

### Measures

#### Sexual Identity Change

The dependent variables in our study were two binary indicators for changes in sexual identity across survey waves. The sexual identity item is a fairly recent addition to the HILDA Survey, having been asked for the first time in 2012 (Wave 12) and every 4 years thereafter (i.e., Waves 16 and 20, in 2016 and 2020, respectively). The question asks, “Which of the following categories best describes how you think of yourself?” Possible responses were (1) Heterosexual or straight, (2) Gay or lesbian, (3) Bisexual, (4) Other, (5) Unsure/don’t know, and (6) Prefer not to say. We recoded response 6 as missing and then created two indicator variables capturing changes in respondents’ sexual identities across consecutive waves (that is, between Waves 12 and 16, and Waves 16 and 20). The first indicator captured changes from a lesbian, gay, bisexual, queer (“other”), or questioning (“unsure/don’t know”) (LGBQ) identity to a straight identity. Respondents scored a 1 on this variable if they identified as LGBQ at time 1 and then identified as heterosexual/straight at time 2. Respondents scored a 0 on this variable of they identified as LGBQ at both time points (including if they changed between LGBQ identities, for example from bisexual to lesbian). The second indicator variable captured changes from a straight identity to an LGBQ identity. Respondents scored a 1 on this variable if they identified as heterosexual/straight at time 1, and then identified as LGBQ at time 2. Respondents scored a 0 on this variable of they identified as heterosexual/straight at both time points. Respondents who did not report their sexual identity in two consecutive waves were missing from both dependent variables, and therefore not included in our analyses. In a robustness check, we re-estimated our main models by dropping individuals who identified as unsure or other (given the uncertainty around the meaning of these responses), and just examined changes between a straight identity and a lesbian, gay, or bisexual (LGB) identity. Results from these robustness checks are available in the Appendix.

#### Conventional Family Attitudes

Our first independent variable was conventional family attitudes, captured using a 6-item index. These items originate from Wave 1 of the Generations and Gender Study (Gauthier et al., 2018) and have been included in the HILDA Survey every 3–4 years since 2005. We used data on these items collected in 2011, 2015, and 2019 (the years immediately prior to those in which sexual identity was measured). The items capture adherence to many of the core tenets of heteronormative family ideology, or what Rubin (1984[2020]) called the “charmed circle.” These attitudes include belief in the importance of marriage (“marriage is a lifetime relationship and should never be ended”), and of raising children in a heterosexual, two-parent family (“children will usually grow up happier if they have a home with both a mother and father”). Responses were measured on a Likert-type scale ranging from 1 (strongly disagree) to 7 (strongly agree). An exploratory factor analysis found that all items loaded on one factor, and the Cronbach’s *α* was an acceptable .75. Items were reverse scored as appropriate, and an overall mean was calculated, with higher scores indicating stronger adherence to conventional family ideology. Respondents with missing data on more than half of the index items were coded as missing. In addition to index scores, we also created a variable capturing change in attitudes from one wave to the next (e.g., from Wave 11 to 15, and Wave 15–19). We created this variable by subtracting score at Time 1 from score at Time 2. As such, a negative score indicates that a person’s endorsement of conventional family ideology decreased, while a positive score indicates that it increased.

#### Preference for Heterosexuality

Our second independent variable was preference for heterosexuality, measured via a single item. This item also originates from the Generations and Gender Study (Gauthier et al., 2018) and was asked in the same years as the conventional family attitudes index. The item asks respondents to rate their level of agreement with the following statement: “Homosexual couples should have the same rights as heterosexual couples do.”^3^ Responses were measured on the same 7-point Likert-type scale described above. For consistency with the other independent variable, we reversed this item so that higher scores indicated higher levels of preference for heterosexuality. It is likely that some of the variation in this item captures respondents’ varying adherence to conventional family ideology. (The correlation between the preference for heterosexuality item and the conventional family attitudes index was *r* = .58.) However, when scores on the family ideology index are entered into the same model and hence controlled for, this item arguably captures respondents’ specific dislike of homosexuality and LGBQ people (i.e., preference for heterosexuality), rather than their beliefs about families. We also created a variable capturing change in preference for heterosexuality from one wave to the next, with a negative score indicating that a person’s level of preference for heterosexuality had decreased, and a positive score indicating that it had increased.

#### Covariates

Covariates were selected based on their previously identified associations with changes in sexual identity and thus potential confounding effects. They included age (Hu & Denier, 2023), gender, educational attainment (Silva & Evans, 2020), geographic location (Campbell, 2023), and changes in circumstances between sexual identity waves, including commencing cohabitation with an other-sex or same-sex partner (Hu & Denier, 2023) and becoming a parent (Campbell, 2023). When our dependent variable was changes to a straight identity, we also controlled for baseline sexual identity (lesbian/gay, bisexual, unsure, or other).

“Age” was a numerical variable capturing participants’ age in years at their last birthday, derived from their self-reported date of birth. “Woman” was a binary indicator for whether participants identified as a woman (in which case they scored a 1 on this variable) or a man (in which case they scored a 0). As previously mentioned, an unfortunate limitation of the HILDA dataset is that it does not contain information on gender diversity, such as participants’ identification as transgender or gender non-binary.

“University degree” was a binary indicator for whether participants had attained a university qualification. It was based on their self-reported highest level of education ever achieved, a question asked in every survey. Participants scored a 1 on this variable if they reported having attained a university qualification including a bachelor’s degree, master’s degree, or doctorate. Participants scored a 0 on this variable if they reported that their highest level of education ever attained was a vocational qualification, high school, or less than high school.

“Lives in a major city” was a binary indicator for whether participants lived in a major city according to the Accessibility/Remoteness Index of Australia Plus Index and based on their household address. Participants scored a 1 on this variable if they resided in an area classified as a major city, and a 0 if they resided in an area classified as inner regional, outer regional, remote or very remote.

“Started living with other-sex partner” and “started living with same-sex partner” were binary indicators derived from information on participants’ household composition at time 2 compared to time 1. Participants scored a 1 on the “started living with other-sex partner” variable if they reported living with an other-sex partner at time 2 but did not report living with an other-sex partner at time 1. Likewise, participants scored a 1 on the “started living with same-sex partner” variable if they reported living with a same-sex partner at time 2 but did not report living with a same-sex partner at time 1.

“Became a parent” was a binary indicator for whether participants became a biological or adoptive parent between time 1 and time 2, based on self-reported household composition. Participants scored a 1 on this variable if they reported residing with at least one child of their own (biological or adoptive) at time 2, but no child of their own at time 1. In all other instances, participants scored a 0 on this variable.

### Analytic Sample and Strategy

To be included in our final analytic sample, respondents needed data from two consecutive waves of sexual identity (2012 and 2016, or 2016 and 2020) in addition to data on conventional family attitudes and preference for heterosexuality from the corresponding waves. Our 2012–2016 sample comprised 10,249 individuals, while our 2016–2020 sample comprised 10,561 individuals. In total, 8323 individuals appeared in both samples, 1926 appeared only in the 2012–2016 sample, and 2238 appeared only in the 2016–2020 sample. For our main analyses, we pooled these data and estimated logistic regression models. Given that many individuals appeared in the data twice (i.e., 2012–2016 sample and 2016–2020 sample), we tested the robustness of our findings in supplementary analyses by estimating random-effects panel regression models. These models account for the nesting of multiple observations within the same individuals and partition the model variance into within-person and between-person components. These tables are available in the appendix. The models we present do not allow us to draw firm conclusions about causality, but they do provide important information about factors which predict sexual identity change.

## Results

### Descriptive Statistics

Between 2012 and 2016, 330 (3.2%) out of 10,249 people changed their sexual identity: 195 (3.5%) out of 5,508 women and 135 (2.8%) out of 4,741 men. Between 2016 and 2020, 430 (4.1%) out of 10,561 people changed their sexual identity: 266 (4.6%) out of 5,754 women and 164 (3.4%) out of 4,807 men. Table 1 shows the number of individuals who changed their sexual identity across each of the two transitions (2012–2016 and 2016–2020) according to their origin and destination identities (i.e., the identities they changed from and to). Individuals who identified as straight were the most likely to report the same identity at the next wave of data collection (~ 98% at both transitions), followed closely by those identifying as lesbian or gay (~ 93% at both transitions). Only 3 individuals identifying as lesbian/gay changed to a straight identity between 2012 and 2016, and only 1 between 2016 and 2020. In contrast, changes to a straight identity were fairly common among individuals identifying as bisexual (30–39%), those reporting an “other” sexual identity (47–67%), and those unsure of their sexual identity (40–47%). Table 5 in the appendix contains transitions matrices for men and women separately and reveals that patterns of sexual identity stability/change were very similar for women and men.

**Table 1:** Sexual identity transition matrices for 2012–2016 and 2016–2020

	Straight	Bisexual	Lesbian/gay	Other	Unsure	Total
	Identity in 2016					
Identity in 2012						
Straight	9,696 (98.2%)	67 (.7%)	12 (.1%)	49 (.5%)	46 (.5%)	9,870
Bisexual	50 (39.4%)	59 (46.5%)	8 (6.3%)	6 (4.7%)	4 (3.2%)	127
Lesbian/gay	3 (2.2%)	3 (2.2%)	130 (93.5%)	1 (.7%)	2 (1.4%)	139
Other	34 (66.7%)	1 (2.0%)	1 (2.0%)	13 (25.5%)	2 (3.9%)	51
Unsure	29 (46.8%)	7 (11.3%)	2 (3.2%)	3 (4.8%)	21 (33.9%)	62
	Identity in 2020					
Identity in 2016						
Straight	9,826 (97.8%)	114 (1.1%)	10 (.1%)	44 (.4%)	58 (.6%)	10,052
Bisexual	56 (29.5%)	107 (56.3%)	10 (5.3%)	14 (7.4%)	3 (1.6%)	190
Lesbian/gay	1 (.6%)	6 (3.7%)	152 (93.3%)	3 (1.8%)	1 (.6%)	163
Other	33 (46.5%)	7 (9.9%)	2 (2.8%)	22 (31.0%)	7 (9.9%)	71
Unsure	34 (40.0%)	14 (16.5%)	3 (3.5%)	10 (11.8%)	24 (28.2%)	85

Data from the Household, Income and Labour Dynamics in Australia Survey.

Table 2 contains descriptive statistics for the 2012–2016 and 2016–2020 samples. In the 2012–2016 sample, 54% were women, 30% had a university degree, and 62% lived in a major city. The mean age was 50.6 years (SD = 17.4). In the 2016–2020 sample, 55% were women, 32% had a university degree, and 62% lived in a major city. The mean age was 50.9 years (SD = 17.8). Mean scores on the conventional family attitudes index dropped slightly across the two samples, from 3.88 (SD = 1.23) in the 2012–2016 sample to 3.61 (SD = 1.28) in the 2016–2020 sample. Mean scores on the preference for heterosexuality item also dropped from 3.42 (SD = 2.26) to 2.73 (SD = 2.13). Within-person changes on the conventional family ideology index were negative and small on average (*M* = − .25 in 2012–2016 and − .22 in 2016–2020); likewise for the preference for heterosexuality item (*M* = − .56 in 2012–2016 and *M* = − .25 in 2016–2020).

**Table 2:** Descriptive statistics for 2012–2016 and 2016–2020 Samples

	LGBQ in 2012		Straight in 2012		2012–2016 Total
	LGBQ in 2016	Straight in 2016	LGBQ in 2016	Straight in 2016	
	*M* (SD)	*M* (SD)	*M* (SD)	*M* (SD)	*M* (SD)
Conventional family attitudes	2.90 (1.37)	3.75 (1.40)	3.75 (1.30)	3.91 (1.22)	3.88 (1.23)
Preference for heterosexuality	1.82 (1.77)	3.29 (2.38)	2.85 (2.27)	3.47 (2.25)	3.42 (2.26)
Change in conventional family attitudes	− .20 (.85)	− .34 (.88)	− .38 (1.16)	− .25 (.83)	− .25 (.85)
Change in preference for heterosexuality	− .24 (1.49)	− .59 (1.87)	− .44 (1.63)	− .57 (1.72)	− .56 (1.72)
Age	45.55 (16.98)	48.09 (19.44)	45.86 (21.6)	50.82 (17.28)	50.57 (17.41)
	*n* (%)	*n* (%)	*n* (%)	*n* (%)	*n* (%)
Woman	140 (53.2%)	79 (68.1%)	96 (55.2%)	5,193 (53.6%)	5508 (53.7%)
Has university degree	94 (35.7%)	27 (23.3%)	37 (21.3%)	2,897 (29.9%)	3055 (29.8%)
Lives in a major city	186 (70.7%)	74 (63.8%)	118 (67.8%)	5,998 (61.9%)	6376 (62.2%)
Started living with other-sex partner	7 (2.7%)	7 (6.0%)	7 (4.0%)	540 (5.6%)	561 (5.5%)
Started living with a same-sex partner	16 (6.1%)	0	4 (2.3%)	0	20 (.2%)
Became a parent	11 (4.2%)	6 (5.2%)	7 (4.0%)	664 (6.9%)	688 (6.7%)
*n*	263	116	174	9,696	10,249

	LGBQ in 2016		Straight in 2016		2016–2020 Total
	LGBQ in 2020	Straight in 2020	LGBQ in 2020	Straight in 2020	
	*M* (SD)	*M* (SD)	*M* (SD)	*M* (SD)	*M* (SD)
Conventional family attitudes	2.63 (1.16)	3.70 (1.35)	3.42 (1.37)	3.65 (1.26)	3.61 (1.28)
Preference for heterosexuality	1.43 (1.22)	2.65 (2.13)	2.34 (2.09)	2.79 (2.14)	2.73 (2.13)
Change in conventional family attitudes	− .25 (.85)	− .05 (1.18)	− .35 (.96)	− .21 (.84)	− .22 (.85)
Change in preference for heterosexuality	− .06 (1.02)	− .01 (2.09)	− .27 (1.87)	− .26 (1.62)	− .25 (1.62)
Age	40.59 (15.96)	50.44 (20.24)	41.12 (19.84)	51.50 (17.55)	50.86 (17.75)
	*n* (%)	*n* (%)	*n* (%)	*n* (%)	*n* (%)
Woman	231 (60.0%)	71 (57.3%)	136 (60.2%)	5,316 (54.1%)	5754 (54.5%)
Has university degree	151 (39.2%)	23 (18.6%)	60 (26.6%)	3,192 (32.5%)	3426 (32.4%)
Lives in a major city	280 (72.7%)	83 (66.9%)	143 (63.3%)	6,017 (61.2%)	6523 (61.8%)
Started living with other-sex partner	25 (6.5%)	9 (7.3%)	7 (3.1%)	528 (5.4%)	569 (5.4%)
Started living with a same-sex partner	18 (4.7%)	0	3 (1.3%)	0	21 (.2%)
Became a parent	16 (4.2%)	6 (4.8%)	12 (5.3%)	632 (6.4%)	666 (6.3%)
*n*	385	124	226	9,826	10,561

Data from the Household, Income and Labour Dynamics in Australia Survey. LGBQ = Lesbian, Gay, Bisexual, Queer, and Questioning

Table 2 also contains descriptive statistics separately according to origin and destination sexual identity group (LGBQ or straight). As Table 2 shows, endorsement of conventional family ideology and preference for heterosexuality were highest in the 2012–2016 sample among those reporting a straight identity at both time points (*M* = 3.91 and 3.47), and lowest among those reporting an LGBQ identity at both time points (*M* = 2.90 and 1.82). In the 2016–2020 sample, endorsement of conventional family attitudes was highest among individuals who changed from an LGBQ to a straight identity (*M* = 3.70), followed closely by individuals who reported a straight identity at both time points (*M* = 3.65). Preference for heterosexuality was also highest among these two groups (*M* = 2.65 and 2.79, respectively). People who consistently identified as LGBQ had the lowest within-person decreases in conventional family attitudes and preference for heterosexuality over time. This finding may seem counterintuitive but must be interpreted in light of their low baseline levels compared to all other groups. There was a strong bivariate association between starting to cohabit with a same-sex partner and maintaining or changing to an LGBQ identity. In both 2012–2016 and 2016–2020, none of the LGBQ-identified individuals who started living with a same-sex partner changed to a straight identity, and all of the straight-identified individuals who started living with a same-sex partner changed to an LGBQ identity.

### Changes to a Straight Identity

We estimated pooled logistic regression models of changes to a straight identity stratified by gender. In the first model for each gender, we entered baseline attitudes and covariates. In the second model, we added changes in attitudes. The results of these models are displayed as odds ratios in Table 3. Commencing cohabitation with a same-sex partner predicted failure perfectly in these models (i.e., none of the LGBQ-identified people who started cohabiting with a same-sex partner changed to a straight identity). Therefore, this variable was automatically omitted from the models resulting in 19 observations not being used in the GBQ women models and 15 observations not being used in the LGBQ men models.

**Table 3:** Results of logistic regression models of change to a straight identity

	OR	95% CI	OR	95% CI
	LGBQ women			
Preference for heterosexuality	.98	.84, 1.13	.96	.80, 1.16
Conventional family attitudes	1.43^**^	1.14, 1.78	1.62^***^	1.25, 2.10
Change in preference for heterosexuality			1.02	.86, 1.22
Change in conventional family attitudes			1.38^*^	1.03, 1.85
Year (ref = 2016)				
2020	.52^**^	.33, .82	.54^**^	.34, .85
Age in years	1.02^**^	1.01, 1.04	1.02^*^	1.00, 1.03
Lives in city	1.15	.70, 1.87	1.14	.70, 1.87
Has university degree	1.11	.64, 1.91	1.17	.68, 2.04
Baseline identity (ref = bisexual)				
Lesbian/gay	.03^***^	.01, .13	.03^***^	.01, .13
Other	1.46	.79, 2.70	1.42	.76, 2.66
Unsure	1.49	.82, 2.70	1.41	.77, 2.60
Started living with other-sex partner	1.24	.57, 2.71	1.22	.56, 2.67
Became a parent	1.28	.50, 3.29	1.38	.53, 3.59
	GBQ men			
Preference for heterosexuality	1.24^*^	1.05, 1.48	1.27^*^	1.02, 1.58
Conventional family attitudes	.93	.71, 1.21	1.01	.74, 1.36
Change in preference for heterosexuality			1.06	.87, 1.31
Change in conventional family attitudes			1.34	.97, 1.85
Year (ref = 2016)				
2020	1.23	.67, 2.25	1.09	.59, 2.02
Age in years	1.01	.99, 1.02	1.00	.99, 1.02
Lives in city	1.09	.58, 2.02	.97	.52, 1.83
Has university degree	.67	.32, 1.38	.67	.32, 1.41
Origin identity (ref = bisexual)				
Lesbian/gay	.03^***^	.01, .12	.03^***^	.01, .13
Other	1.96	.92, 4.15	1.98	.93, 4.23
Unsure	.75	.38, 1.50	.72	.36, 1.46
Started living with other-sex partner	1.06	.25, 4.44	.99	.24, 4.10
Became a parent	.82	.15, 4.41	.81	.15, 4.50

Data from the Household, Income and Labour Dynamics in Australia Survey. LGBQ = Lesbian, Gay, Bisexual, Queer, and Questioning. LGBQ women = 502 observations from 401 individuals. GBQ men = 352 observations from 267 individuals. Variable “started living with same-sex partner” predicted failure perfectly and was therefore automatically omitted from the models. ^*^ *p* < .05, ^**^ *p* < .01, ^***^ *p* < .001

In the first model for LGBQ women, we found a positive association between endorsement of conventional family ideology and the odds of changing to a straight identity. For every one-point increase in baseline scores on this index, the odds of changing to a straight identity increased by a factor of 1.43 (*p* = .002). There was no association between preference for heterosexuality scores and the propensity to change to a straight identity. Among LGBQ women, the odds of changing to a straight identity increased with age and were lower in 2016–2020 than 2012–2016. Consistent with the descriptive trends previously described, the odds of changing to a straight identity were similarly high for women identifying as bisexual, other, or unsure, but substantially smaller for women identifying as lesbian. When changes in attitudes were added in the second model, we found that baseline levels of conventional family attitudes remained a significant, positive predictor of changing to a straight identity for LGBQ women. In addition, within-person increases in the endorsement of conventional family attitudes were associated with increased odds of changing to a straight identity, controlling for baseline attitudes.

In the first model for GBQ men, we found no association between endorsement of conventional family attitudes and the odds of changing to a straight identity. Instead, there was a positive association between preference for heterosexuality and the propensity to change to a straight identity. For every one-point increase in baseline preference for heterosexuality levels, the odds of changing from an LGBQ to a straight identity increased by a factor of 1.24 (*p* = .014). The only other significant predictor in this model was origin sexual identity. The odds of changing to a straight identity were similar for men identifying as bisexual, other, or unsure, but substantially lower for men identifying as gay (compared to bisexual). This pattern of findings did not change in the second model for GBQ men, with neither changes in conventional family attitudes nor changes in preference for heterosexuality emerging as significant predictors of changing to a straight identity. Altogether, our findings for changes to a straight identity were consistent with our expectations, with endorsement of conventional family ideology a stronger “push” factor for LGBQ women and preference for heterosexuality a stronger “push” factor for GBQ men.

### Changes to an LGBQ Identity

Following the same strategy used to examine changes to a straight identity, we estimated gender-stratified logistic regression models to examine changes to an LGBQ identity. In the first model for each gender, we entered baseline attitudes and covariates. In the second model, we added changes in attitudes. The results of these models are displayed as odds ratios in Table 4. Commencing cohabitation with a same-sex partner predicted success perfectly in these models (i.e., all of the straight-identified people who started cohabiting with a same-sex partner changed to an LGBQ identity). Therefore, this variable was automatically omitted from the models resulting in 6 observations not being used in the straight women models and 1 observation not being used in the straight men models.

**Table 4:** Results of logistic regression models of change to an LGBQ identity

	OR	95% CI	OR	95% CI
	Straight women			
Preference for heterosexuality	.95	.87, 1.04	.95	.85, 1.06
Conventional family attitudes	.93	.81, 1.06	.88	.75, 1.03
Change in preference for heterosexuality			.96	.86, 1.08
Change in conventional family attitudes			.84	.70, 1.00
Year (ref = 2016)				
2020	1.38^*^	1.05, 1.81	1.37^*^	1.05, 1.80
Age in years	.96^***^	.96, .97	.97^***^	.96, .97
Lives in city	1.30	.98, 1.73	1.30	.98, 1.74
Has university degree	.58^***^	.43, .78	.57^***^	.42, .78
Started living with other-sex partner	.45^*^	.24, .87	.46^*^	.24, .89
Became a parent	.69	.40, 1.21	.69	.40, 1.21
	Straight men			
Preference for heterosexuality	.94	.86, 1.02	.94	.85, 1.04
Conventional family attitudes	.99	.85, 1.16	.96	.81, 1.14
Change in preference for heterosexuality			.99	.89, 1.10
Change in conventional family attitudes			.90	.74, 1.09
Year (ref = 2016)				
2020	1.13	.83, 1.54	1.13	.83, 1.55
Age in years	.98^***^	.97, .99	.98^***^	.97, .99
Lives in city	1.02	.74, 1.42	1.03	.74, 1.42
Has university degree	.59^**^	.40, .88	.59^**^	.40, .87
Started living with other-sex partner	.33^*^	.12, .91	.33^*^	.12, .91
Became a parent	.40^*^	.16, .98	.40^*^	.16, .98

Data from the Household, Income and Labour Dynamics in Australia Survey. Straight women = 10,735 observations from 6,400 individuals. Straight men = 9,180 observations from 5,611 individuals. Variable “started living with same-sex partner” predicted success perfectly and was therefore automatically omitted from the models. ^*^ *p* < .05, ^**^ *p* < .01, ^***^ *p* < .001

In the first model for straight women, we found no association between preference for heterosexuality or the endorsement of conventional family attitudes and the odds of changing to an LGBQ identity. This pattern did not change in the second model for straight women, with neither changes in preference for heterosexuality nor changes in conventional family attitudes associated with the propensity to change to an LGBQ identity. The odds of changing to an LGBQ identity decreased significantly with age and were higher in 2016–2020 than 2012–2016. This finding is the inverse of the pattern observed for changes to a straight identity (which increased with age and were less common in 2016–2020) and is consistent with well-documented trends of increasing LGBQ identification among more recent birth cohorts (Jones, 2025)—especially among women (England et al., 2016). Straight women were significantly less likely to change to an LGBQ identity if they had a university degree (compared to none) or started cohabiting with an other-sex partner.

Results from the first model for straight men were very similar to those for straight women: We found no association between endorsement of conventional family attitudes or preference for heterosexuality and the odds of changing to an LGBQ identity. As for women, the odds of changing to an LGBQ identity decreased significantly with age for straight men. Likewise, straight men were less likely to change to an LGBQ identity if they had a university degree (compared to none), started cohabiting with an other-sex partner, or became a parent. When changes in attitudes were added in the second model, we found no significant associations between within-person changes in the endorsement of conventional family attitudes or preference for heterosexuality and the propensity to change to an LGBQ identity.

### Robustness Checks

We conducted supplementary analyses to test the robustness of our findings. First, we re-estimated our models by only including individuals who identified as straight, lesbian/gay, or bisexual, and dropping those who had responded to the sexual identity item with “other” and “unsure” (given that we don’t know for certain what these responses meant). The results for changes to a straight identity are presented in Table 6 in the appendix and were highly consistent with results from our main models. For LGB women, higher endorsement of conventional family attitudes at baseline and increases in these attitudes over time were associated with increased odds of changing to a straight identity. For LGB men, higher levels of preference for heterosexuality at baseline were associated with increased odds of changing to a straight identity. Overall, these findings suggest that the predictors of changing to a straight identity are robust to alternative measures of sexual identification, and specifically whether “other” and “unsure/don’t know” responses are combined with LGB responses.

For changes to an LGB identity, results diverged somewhat from our main models (see Table 7 in the appendix). Baselines levels of preference for heterosexuality were a significant, negative predictor of changing to an LGB identity for straight women (which was not the case when “other” and “unsure/don’t know” responses were combined with LGB responses, as in main models). This finding suggests that higher levels of internalized preference for heterosexuality reduce the likelihood of straight-identified women changing to a lesbian or bisexual identity, but perhaps not the odds of changing to an unsure and/or “other” identity.

In another robustness check, we re-estimated our main models as random-effects panel regression models rather than pooled regression models. Random-effects panel regression models account for the nesting of observations within individuals and partition the variance into between-individual versus within-individual components. The results of the panel regression models are presented in Appendix Tables 8 and 9 and were highly consistent with the results of our main models.

Last, we combined data for women and men and re-estimated our main models with interactions between each explanatory variable and gender. Results from these interaction models are displayed in Appendix Tables 10 and 11. Consistent with the results from our gender-stratified models, we found a significant interaction between being a woman and preference for heterosexuality (OR .79, *p* = .039), and between being a woman and endorsement of conventional family attitudes (OR 1.53, *p* = .015), when predicting changes from an LGBQ identity to a straight identity. Also consistent with the results of our stratified models, we found no significant interactions between gender and endorsement of conventional family attitudes or preference for heterosexuality when predicting changes to an LGBQ identity.

## Discussion

In this study, we used a large, nationally representative survey of Australia to examine factors associated with changes in sexual identity. Overall, we observed rates of sexual identity change in the order of 3–4%, which is similar to other studies that used adult samples (Charlton et al., 2016, Dickson et al., 2013, Mock & Eibach, 2012; see Silva, 2025 for a discussion of national context). Consistent with evidence that LGBQ identification is increasing over time, especially among women and members of the Millennial and Gen Z cohorts (England et al., 2016; Jones, 2025), we found that changes to an LGBQ identity increased with time among women and decreased with age for both genders. We also found that changes in family/relationship circumstances were associated with changes in sexual identity, which is again consistent with prior research (Hu & Denier, 2023). Among straight men and women, those who started living with an other-sex partner were significantly less likely to change to an LGBQ identity, while all straight-identified people who started cohabiting with a same-sex partner changed to an LGBQ identity. Straight men who became a father were significantly less likely to change to an LGBQ identity.

Most changes from a straight identity were to bisexual, other, or unsure, rather than lesbian or gay identities. Likewise, most changes to straight were from bisexual, other, or unsure, with very few lesbian or gay people changing to a straight identity.^4^ This pattern is consistent with prior research (Campbell et al., 2021; Hu & Denier, 2023; Mittleman, 2023a; Ott, 2011; Savin-Williams et al., 2012) and could reflect that most people who identify as bisexual, other, or unsure report some attraction to people of the other sex, whereas most people who identify as gay or lesbian do not (Fu et al., 2019; Mishel, 2019). This pattern provides important context for interpreting our main results described in the next section: While heteronormativity impacts all LGBQ people, it may be more likely to act as a push factor toward straight identification for plurisexual and questioning people than for lesbian or gay people.^5^More generally, the rarity of changes to or from a gay/lesbian identity shows that the findings are applicable primarily to people who changed to or from bisexual, unsure, or other identities. In Australia, as in other countries, monosexual identities (heterosexual, gay/lesbian) are on average more likely to stay the same between points in time than plurisexual or open-ended identities such as bisexual, unsure, or other.

### Heteronormativity and Shifts to a Straight Identity

The main goal of our study was to examine associations between internalized heteronormativity and the propensity to change sexual identity among women versus men. We found that the attitudinal predictors of changing from an LGBQ to a straight identity were gendered in line with our expectations. Endorsement of conventional family ideology was positively associated with the odds of changing to a straight identity among LGBQ women but not men, suggesting that traditional attitudes about family are a stronger push factor for changing to a straight identity among women. In our introduction, we argued that same-sex behavior is permitted among women when it is perceived as complementing rather than replacing heterosexuality (Rupp et al., 2014). Examples of such behavior include threesomes between one man and two women, and “straight girls kissing” at college parties (Diamond, 2005; Pham, 2019; Yost & McCarthy, 2012). This greater social acceptance is arguably a key factor in the higher rates of LGBQ (especially bisexual) identification among women compared to men. Yet, as we also argued in our introduction, this acceptance comes with many conditions. Feminist scholars have long argued that the heterosexual nuclear family and the ideology that supports and upholds it were designed to control women and ensure men’s access to their labor (e.g., Rich, 1980 [2003]). Our results suggest that the internalization of this ideology acts as a constraint on women’s sexualities. Thus, while (young) women might feel increasingly free to reject straightness in favor of mostly heterosexual, bisexual, queer, and questioning identities (Perales et al., 2021), our findings suggest that endorsement of conventional family values could ultimately prompt some women to shift back.

In contrast, preference for heterosexuality was positively associated with the odds of changing to a straight identity among GBQ men but not LGBQ women. While a certain degree of bisexual behavior among young women is somewhat socially accepted, the same is not the case for bisexual behavior among young men. A recent meta-analysis showed that men are especially negative toward men's bisexuality (Manalastas et al., 2025). Men’s same-sex behavior was shown to elicit disgust and lead to antigay hostility in a sample of heterosexual men (Ray & Parkhill, 2021), and homophobic bullying remains central to the enactment and enforcement of normative masculinity (Mittleman, 2023b). Our findings suggest that this preference for heterosexuality can act as a constraint on men who might otherwise identify as bisexual, queer, or questioning, pushing them back toward a normative straight identity.

### Heteronormativity and Shifts to an LGBQ Identity

Interestingly, the attitudinal predictors of changing from a straight to an LGBQ identity were not gendered in the same way as the predictors of changing to a straight identity. In fact, there were no significant attitudinal predictors of changing to an LGBQ identity in our main models. This diverges from the recent findings of Lilly et al. (2024), who found lower odds of changing from a heterosexual to a plurisexual identity among people reporting higher levels of religiosity and political conservatism. Given that most (albeit not all) people who identify as straight do not report same-sex attractions or engage in same-sex behavior (Fu et al., 2019; Mishel, 2019; Richters et al., 2014; Silva & Fetner, 2022), our results could reflect that internalized heteronormativity has little effect on the propensity to change to an LGBQ identity among straight-identified people with little capacity for non-heterosexuality to begin with. Future research should incorporate information on sexual attractions and behavior to investigate whether there is a subset of straight-identified people with an apparent capacity for non-heterosexuality for whom internalized heteronormativity does play a role in shaping their sexual identities. The results of our supplementary analyses further suggest the importance of distinguishing between different destination identities (e.g., changing from straight to bisexual versus questioning) in any such endeavor.

### Conclusion

Using data from a large, nationally representative survey, our study is one of just a few internationally to have examined gendered predictors of sexual identity change. A key takeaway from this project is that conventional family ideology is a stronger predictor of changing to a straight identity for LGBQ-identified women than men. Thus, even as increasing numbers of women opt out of marriage and childrearing, and bisexual identification is on the rise, conventional family ideology continues to exert pressure on women’s lives. How this pattern impacts women’s long-term relationship and well-being trajectories is a topic deserving attention. Meanwhile, preference for heterosexuality is a predictor of changing to a straight identity for GBQ-identified men only, and the long-term impacts of this dynamic—on bisexual men, in particular—should also be explored.

## Appendix

See Tables

**Table 5:** Sexual identity transition matrices for women and men

Destination identity						
	Straight	Bisexual	Lesbian/gay	Other	Unsure	Total
*Women*						
Origin identity						
Straight	10,509 (97.8%)	122 (1.1%)	6 (.1%)	43 (.4%)	61 (.6%)	10,741
Bisexual	69 (31.2%)	123 (55.7%)	11 (5.0%)	14 (6.3%)	4 (1.8%)	221
Lesbian/gay	2 (1.4%)	6 (4.2%)	128 (89.5%)	4 (2.8%)	3 (2.1%)	143
Other	38 (51.4%)	6 (8.1%)	3 (4.1%)	23 (31.1%)	4 (5.4%)	74
Unsure	41 (49.4%)	15 (18.1%)	2 (2.1%)	7 (8.4%)	18 (21.7%)	83
*Men*						
Origin identity						
Straight	9,013 (98.2%)	59 (.6%)	16 (.2%)	50 (.5%)	43 (.5%)	9,181
Bisexual	37 (38.5%)	43 (44.8%)	7 (7.3%)	6 (6.3%)	3 (3.1%)	96
Lesbian/Gay	2 (1.3%)	3 (1.9%)	154 (96.9%)	0	0	159
Other	29 (60.4%)	2 (4.2%)	0	12 (25.0%)	5 (10.4%)	48
Unsure	22 (34.4%)	6 (9.4%)	3 (4.7%)	6 (9.4%)	27 (42.2%)	64

Data from the Household, Income and Labour Dynamics in Australia Survey, 2012–2016 and 2016–2020 pooled

5,

**Table 6:** Results of logistic regression models of change to straight identity from LGB

	OR	95% CI	OR	95% CI
	LGB women			
Preference for heterosexuality	1.21	.94, 1.56	1.36	.93, 1.99
Conventional family attitudes	1.59^**^	1.12, 2.24	2.03^***^	1.34, 3.08
Change in preference for heterosexuality			1.17	.84, 1.63
Change in conventional family attitudes			1.96^**^	1.23, 3.12
Year (ref = 2016)				
2020	.59	.30, 1.13	.62	.31, 1.22
Age in years	1.03^**^	1.01, 1.06	1.03^*^	1.01, 1.06
Lives in city	1.54	.76, 3.15	1.60	.76, 3.37
Has university degree	1.54	.72, 3.28	1.62	.74, 3.56
Baseline identity (ref = bisexual)				
Lesbian/gay	.03^***^	.01, .12	.02^***^	.01, .12
Started living with other-sex partner	2.28	.87, 5.95	1.96	.74, 5.24
Became a parent	1.80	.50, 6.41	2.24	.61, 8.25
	GB men			
Preference for heterosexuality	1.53^**^	1.15, 2.03	2.04^**^	1.30, 3.19
Conventional family attitudes	1.17	.77, 1.78	1.20	.73, 1.98
Change in preference for heterosexuality			1.53	.99, 2.36
Change in heteronormativity family attitudes			1.69	1.00, 2.86
Year (ref = 2016)				
2020	2.86	.93, 8.75	1.81	.56, 5.84
Age in years	1.03^*^	1.00, 1.06	1.03	1.00, 1.05
Lives in city	.75	.27, 2.14	.46	.15, 1.40
Has university degree	.57	.18, 1.75	.59	.18, 2.02
Origin identity (ref = bisexual)				
Lesbian/gay	.03^***^	.01, .15	.03^***^	.01, .15
Started living with other-sex partner	21.92	.51, 948.37	10.05	.38, 267.92
Became a parent	23.58	0.12, 4769.45	10.00	.00, 20,893.79

Data from the Household, Income and Labour Dynamics in Australia Survey. LGB = Lesbian, Gay, and Bisexual. LGB women = 323 observations from 248 individuals. GB men = 231 observations from 163 individuals. Variable “started living with same-sex partner” predicted failure perfectly and was therefore automatically omitted from the models. ^*^ *p* < .05, ^**^ *p* < .01, ^***^ *p* < .001

^6^,

**Table 7:** Results of logistic regression models of change to an LGB identity

	OR	95% CI	OR	95% CI
	Straight women			
Preference for heterosexuality	.83^*^	.71, .96	.79^*^	.65, .96
Conventional family attitudes	.88	.73, 1.07	.80	.63, 1.00
Change in preference for heterosexuality			.86	.71, 1.05
Change in conventional family attitudes			.73^*^	.57, .94
Year (ref = 2016)				
2020	1.55^*^	1.06, 2.25	1.54^*^	1.06, 2.23
Age in years	.94^***^	.93, .95	.94^***^	.93, .96
Lives in city	1.24	.84, 1.83	1.24	.84, 1.83
Has university degree	.52^**^	.34, .79	.51^**^	.34, .78
Started living with other-sex partner	.43^*^	.20, .94	.45^*^	.21, .98
Became a parent	.87	.46, 1.65	.87	.46, 1.63
	Straight men			
Preference for heterosexuality	.87	.76, 1.00	.86	.73, 1.02
Conventional family attitudes	.91	.73, 1.15	.87	.67, 1.13
Change in preference for heterosexuality			.94	.79, 1.12
Change in conventional family attitudes			.85	.63, 1.14
Year (ref = 2016)				
2020	1.35	.84, 2.16	1.36	.84, 2.18
Age in years	.96^***^	.94, .97	.96^***^	.94, .97
Lives in city	1.10	.67, 1.81	1.10	.67, 1.80
Has university degree	.64	.36, 1.13	.63	.35, 1.11
Started living with other-sex partner	.13^*^	.02, .96	.13^*^	.02, .95
Became a parent	.47	.15, 1.51	.47	.15, 1.51

Data from the Household, Income and Labour Dynamics in Australia Survey. Straight women = 10,632 observations from 6,331 individuals. Straight men = 9,087 observations from 5,543 individuals. Variable “started living with same-sex partner” predicted success perfectly and was therefore automatically omitted from the models. ^*^ *p* < .05, ^**^ *p* < .01, ^***^ *p* < .001

^7^,

**Table 8:** Results of random-effects logistic regression models of change to straight identity from LGBQ

	OR	95% CI	OR	95% CI
	LGBQ women			
Preference for heterosexuality	.97	.72, 1.31	.97	.63, 1.48
Conventional family attitudes	1.89^*^	1.13, 3.18	2.80^*^	1.27, 6.18
Change in preference for heterosexuality			1.10	.75, 1.61
Change in conventional family attitudes			2.02	.99, 4.13
Year (ref = 2016)				
2020	.45	.20, 1.01	.51	.20, 1.29
Age in years	1.05^*^	1.01, 1.10	1.05^*^	1.00, 1.11
Lives in city	1.27	.48, 3.36	1.27	.42, 3.87
Has university degree	1.04	.35, 3.03	1.16	.34, 4.01
Baseline identity (ref = bisexual)				
Lesbian/gay	.00^**^	.00, .11	.00^**^	.00, .11
Other	1.56	.44, 5.48	1.46	.34, 6.19
Unsure	1.79	.54, 5.96	1.60	.40, 6.40
Started living with other-sex partner	1.55	.36, 6.67	1.55	.30, 8.00
Became a parent	.86	.13, 5.64	.76	.09, 6.81
	GBQ men			
Preference for heterosexuality	1.26^*^	1.01, 1.56	1.32	.97, 1.79
Conventional family attitudes	.92	.69, 1.23	1.00	.71, 1.41
Change in preference for heterosexuality			1.09	.85, 1.39
Change in heteronormativity family attitudes			1.39	.94, 2.04
Year (ref = 2016)				
2020	1.26	.63, 2.52	1.14	.55, 2.36
Age in years	1.01	.99, 1.03	1.00	.99, 1.02
Lives in city	1.09	.56, 2.11	.95	.46, 1.97
Has university degree	.64	.27, 1.51	.63	.26, 1.56
Origin identity (ref = bisexual)				
Lesbian/gay	.03^***^	.00, .17	.02^***^	.00, .19
Other	2.02	.86, 4.77	2.12	.84, 5.33
Unsure	.74	.34, 1.58	.69	.30, 1.58
Started living with other-sex partner	1.07	.23, 4.89	1.00	.20, 4.92
Became a parent	.80	.14, 4.77	.78	.12, 5.24

Data from the Household, Income and Labour Dynamics in Australia Survey. LGBQ = Lesbian, Gay, Bisexual, Queer, and Questioning. LGBQ women = 502 observations from 401 individuals. GBQ men = 352 observations from 267 individuals. Variable “started living with same-sex partner” predicted failure perfectly and was therefore automatically omitted from the models. ^*^ *p* < .05, ^**^ *p* < .01, ^***^ *p* < .001

^8^,

**Table 9:** Results of random-effects logistic regression models of change to an LGBQ identity

	OR	95% CI	OR	95% CI
	Straight women			
Preference for heterosexuality	.95	.87, 1.04	.95	.85, 1.06
Conventional family attitudes	.93	.81, 1.06	.88	.75, 1.03
Change in preference for heterosexuality			.96	.86, 1.08
Change in conventional family attitudes			.84	.70, 1.00
Year (ref = 2016)			1.00	1.00, 1.00
2020	1.38^*^	1.05, 1.81	1.37^*^	1.05, 1.80
Age in years	.96^***^	.96, .97	.97^***^	.96, .97
Lives in city	1.30	.98, 1.73	1.30	.98, 1.74
Has university degree	.58^***^	.43, .78	.57^***^	.42, .78
Started living with other-sex partner	.45^*^	.24, .87	.46^*^	.24, .89
Became a parent	.69	.40, 1.21	.69	.40, 1.21
	Straight men			
Preference for heterosexuality	.91	.80, 1.04	.93	.79, 1.10
Conventional family attitudes	.96	.76, 1.23	.90	.68, 1.19
Change in preference for heterosexuality			1.01	.86, 1.18
Change in conventional family attitudes			.85	.63, 1.15
Year (ref = 2016)				
2020	1.73^*^	1.09, 2.73	1.76^*^	1.11, 2.80
Age in years	.97^***^	.96, .99	.97^**^	.96, .99
Lives in city	1.00	.59, 1.72	1.01	.58, 1.74
Has university degree	.45^*^	.23, .87	.44^*^	.23, .86
Started living with other-sex partner	.19^*^	.04, .83	.18^*^	.04, .82
Became a parent	.28	.08, 1.01	.28	.08, 1.03

Data from the Household, Income and Labour Dynamics in Australia Survey. Straight women = 10,735 observations from 6,400 individuals. Straight men = 9,180 observations from 5,611 individuals. Variable “started living with same-sex partner” predicted success perfectly and was therefore automatically omitted from the models. ^*^ *p* < .05, ^**^ *p* < .01, ^***^ *p* < .001

^9^,

**Table 10:** Interaction terms from logistic regression model of change to straight identity from LGBQ

	OR	95% CI	OR	95% CI
*LGBQ women and men*				
Woman × Preference for heterosexuality	.79^*^	.63, .99	.76	.57, 1.01
Woman × Conventional family attitudes	1.53^*^	1.09, 2.16	1.61^*^	1.08, 2.40
Woman × Change in preference for heterosexuality			.96	.73, 1.26
Woman × Change in conventional family attitudes			1.03	.67, 1.59
Woman × Year 2020	.42^*^	.20, .90	.49	.23, 1.07
Woman × Age in years	1.01	.99, 1.04	1.01	.99, 1.04
Woman × Lives in city	1.06	.48, 2.33	1.18	.53, 2.62
Woman × Has university degree	1.66	.67, 4.12	1.75	.69, 4.41
Woman × Baseline identity (ref = bisexual)				
Woman × Lesbian/gay	1.05	.13, 8.40	1.06	.13, 8.47
Woman × Other	.75	.28, 1.97	.72	.27, 1.92
Woman × Unsure	1.98	.80, 4.93	1.96	.77, 4.96
Woman × Started living with other-sex partner	1.18	.23, 6.05	1.24	.24, 6.29
Woman × Became a parent	1.57	.23, 10.82	1.70	.24, 12.04

Data from the Household, Income and Labour Dynamics in Australia Survey. Observations = 854, from 668 individuals. Variable “started living with same-sex partner” predicted failure perfectly and was therefore automatically omitted from the models. Main effects for these models are equal to the coefficients for LGBQ men shown in Table 3. Therefore, they are not shown again here. ^*^ *p* < .05, ^**^ *p* < .01, ^***^ *p* < .001

^10^, and

**Table 11:** Interaction terms from logistic regression model of change to an LGBQ identity

	OR	95% CI	OR	95% CI
*Straight women and men*				
Woman × Preference for heterosexuality	1.01	.89, 1.14	1.01	0.87, 1.17
Woman × Conventional family attitudes	.93	.76, 1.14	.92	0.73, 1.15
Woman × Change in preference for heterosexuality			.97	0.83, 1.13
Woman × Change in conventional family attitudes			.93	0.71, 1.22
Woman × Year 2020	1.22	.81, 1.85	1.21	0.80, 1.84
Woman × Age in years	.98^**^	.97, 1.00	.98^*^	0.97, 1.00
Woman × Lives in city	1.27	.82, 1.96	1.27	0.82, 1.96
Woman × Has university degree	.97	.59, 1.59	.97	0.59, 1.60
Woman × Started living with other-sex partner	1.37	.41, 4.53	1.40	0.42, 4.66
Woman × Became a parent	1.74	.61, 5.01	1.74	0.60, 4.99

Data from the Household, Income and Labour Dynamics in Australia Survey. Observations = 19,719 from 11,874 individuals. Variable “started living with same-sex partner” predicted success perfectly and was therefore automatically omitted from the models. Main effects for these models are equal to the coefficients for straight men shown in Table 4. Therefore, they are not shown again here. ^*^ *p* < .05, ^**^ *p* < .01, ^***^ *p* < .001

^11^.

## Footnotes

1 See also van Anders (2015) for a discussion of how individuals may experience “branching,” that is, attractions and/or behaviors that others may perceive as not aligning with their sexual identity (e.g., someone who identifies as heterosexual and experiences same-sex attractions).

2 Although the term “homosexual” is considered outdated in some Anglophone contexts today, it is one of the only terms that translates similarly across languages.

3 Unfortunately, HILDA contained only one item which gauged attitudes toward LGB people. Future research should include additional measures to more comprehensively examine the relationship between sexual identity change and attitudes toward LGB people. Some of these measures could be phrased to measure concepts apart from same-sex couples, such as whether LGB people should be accepted by society, to ensure that there is less conceptual overlap between variables gauging support for LGB people and conventional family structures.

4 For some people, changes to sexual identities between waves could come after shifts to behavior and/or attraction. Thus, identity change could reflect the adoption of identities to be in “alignment” with attractions or behaviors which had changed.

5 In the USA, most of the increase in LGBQ identification is attributable to young women who identify as bisexual (Jones, 2025). Young bisexual-identified women today are much less likely to have had a same-sex partner in the past five years than young bisexual-identified women through 2014 (Kaufmann, 2022). While trends for Australia are unknown because of data limitations, it is possible that some Australian women who once identified as bisexual began to identify as heterosexual due to their current/recent sexual behavior with men.
